# Myosin IIA Modulates T Cell Receptor Transport and CasL Phosphorylation during Early Immunological Synapse Formation

**DOI:** 10.1371/journal.pone.0030704

**Published:** 2012-02-08

**Authors:** Yan Yu, Nicole C. Fay, Alexander A. Smoligovets, Hung-Jen Wu, Jay T. Groves

**Affiliations:** 1 Howard Hughes Medical Institute, Department of Chemistry, University of California, Berkeley, California, United States of America; 2 Department of Molecular and Cell Biology, University of California, Berkeley, California, United States of America; 3 Physical Biosciences Division, Lawrence Berkeley National Laboratory, Berkeley, California, United States of America; 4 Mechanobiology Institute, National University of Singapore, Singapore, Singapore; University Paris Sud, France

## Abstract

Activation of T cell receptor (TCR) by antigens occurs in concert with an elaborate multi-scale spatial reorganization of proteins at the immunological synapse, the junction between a T cell and an antigen-presenting cell (APC). The directed movement of molecules, which intrinsically requires physical forces, is known to modulate biochemical signaling. It remains unclear, however, if mechanical forces exert any direct influence on the signaling cascades. We use T cells from AND transgenic mice expressing TCRs specific to the moth cytochrome *c* 88–103 peptide, and replace the APC with a synthetic supported lipid membrane. Through a series of high spatiotemporal molecular tracking studies in live T cells, we demonstrate that the molecular motor, non-muscle myosin IIA, transiently drives TCR transport during the first one to two minutes of immunological synapse formation. Myosin inhibition reduces calcium influx and colocalization of active ZAP-70 (zeta-chain associated protein kinase 70) with TCR, revealing an influence on signaling activity. More tellingly, its inhibition also significantly reduces phosphorylation of the mechanosensing protein CasL (Crk-associated substrate the lymphocyte type), raising the possibility of a direct mechanical mechanism of signal modulation involving CasL.

## Introduction

The spatial organization of cell membrane receptors at intercellular junctions is emerging as an important aspect of many signal transduction processes [1]–[5]. One paradigmatic example is T cell activation in which T cell receptors (TCRs) engage their ligands, antigenic peptide loaded major histocompatibility complex proteins (pMHC), on the surface of antigen-presenting cells (APCs). This cell-cell junction, known as the immunological synapse (IS), exhibits an elaborately choreographed spatial reorganization of proteins on multiple length scales, ranging from molecular dimensions to the size of the cell itself [6], [7]. Upon the triggering, T cell receptors (TCRs) collectively nucleate into microclusters of tens to hundreds of molecules together with kinases and adaptor proteins. The signaling clusters are subsequently transported centripetally, ultimately accumulating in the central supramolecular activating complex (cSMAC) where signaling is attenuated [8]–[11]. Meanwhile, integrins reorganize into a ring structure, forming the peripheral supramolecular activating complex (pSMAC). Interference with protein pattern formation by physically imposed barriers to TCR translocation leads to changes in TCR phosphorylation, duration and magnitude of calcium response, as well as changes in T cell triggering thresholds [12]–[14].

In the terminology of thermodynamics, force is the conjugate variable to space. As such, spatial organization and mechanical forces are intrinsically coupled; in general, one doesn't occur without the other. In the case of the immunological synapse, forces have been implicated in its formation since its initial identification [15]. Retrograde flow of the actin cytoskeleton drives segregation of signaling complexes at the IS and is required for sustaining TCR signaling [16]–[21]. Dynein has also been shown in a recent study to drive microtubule-dependent transport of TCRs and to negatively regulate T cell signaling [22]. In the immunological synapse, the role of non-muscle myosin IIA, the myosin II isoform that is dominantly expressed in T cells, has been debated in several studies [15], [23], [24], but without consensus.

Here we examine the role of myosin IIA in the formation of the immunological synapse by tracking movements of TCRs, actin, and myosin with high spatial and temporal resolution. Primary T cells are activated by pMHC and inter-cellular adhesion molecule (ICAM) −1, both of which are tethered to supported lipid bilayers by polyhistidine/nickel-chelating lipid linkages. Both proteins, freely mobile in the supported bilayer, readily assemble into microclusters and larger scale organization in response to driving forces applied by the cell. This hybrid live cell – supported membrane junction enables high resolution imaging of the immunological synapse using total internal reflection fluorescence (TIRF) microscopy [25]. By analyzing movements of TCRs, actin, and myosin, we demonstrate that myosin IIA makes a distinctive contribution to TCR cluster movement during the first one to two minutes after T cell stimulation. Inhibition with blebbistatin or ML-7 reduces both calcium influx and spatial colocalization of active ZAP-70 with TCR microclusters. Thus myosin IIA contributes, at least indirectly, to TCR signaling. A more telling observation is that myosin inhibition also reduces phosphorylation of the mechanosensing protein CasL (Crk-associated substrate the lymphocyte type), raising the hypothesis of a direct mechanical mechanism of signal modulation involving CasL.

## Results

### Myosin IIA transiently drives translocation of TCR microclusters

**Figure 1:**
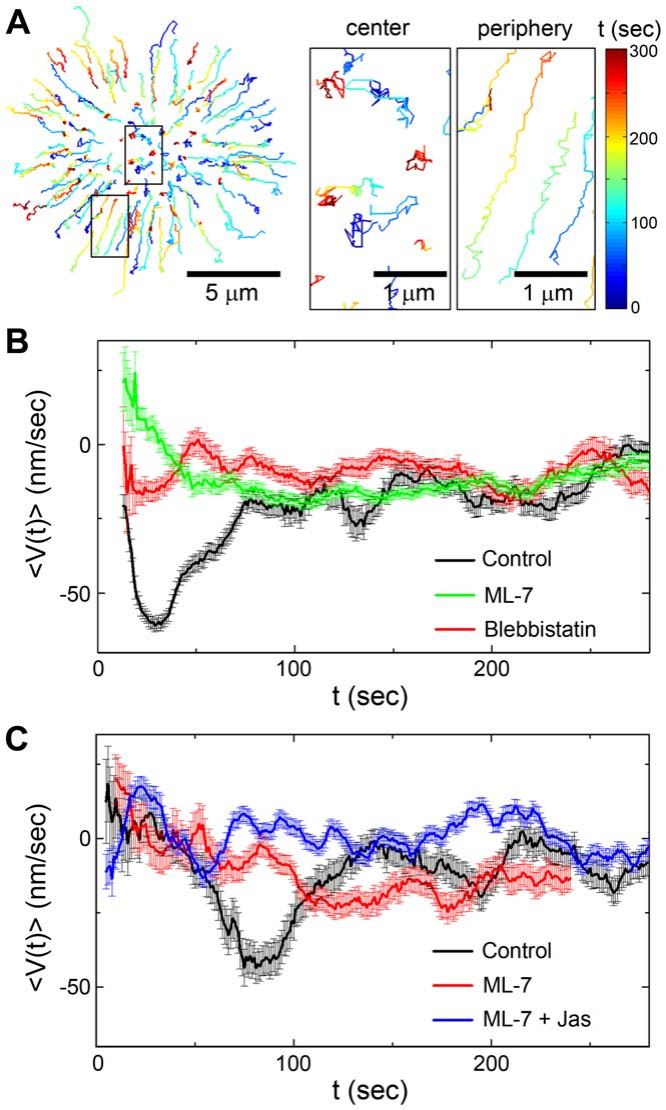
Myosin IIA transiently drives TCR translocation and actin retrograde flow in immunological synapse formation. T cell receptors (TCRs) were labeled with H57 αTCR F_ab_ (Alexa Fluor dyes) and imaged starting from the initial cell-bilayer contact (*t* = 0 sec). (A) Trajectories of all TCR microclusters (Alexa Fluor 594) show their centripetal movement at the cell periphery and highly confined motion at the central area of the immunological synapse. Color bar corresponds to the elapsed time after the initial cell-bilayer contact. Data are representative of 6 independent experiments. (B) Inhibition of myosin IIA changes the time-dependence of TCR microcluster translocation. Time-averaged radial velocities (<V(*t*)>) of TCRs (Alexa Fluor 643) in individual cells are plotted against the elapsed time after the initial cell-bilayer contact in the presence of DMSO control, blebbistatin, or ML-7. Data are representative of 5 independent experiments. (C) Inhibition of myosin IIA changes the time-dependence of actin retrograde flow during the immunological synapse formation. Time-averaged radial velocities (<V(*t*)>) of tracked EGFP-UtrCH in individual cells are plotted against the elapsed time after the initial cell-bilayer contact in the presence of DMSO control, ML-7, or ML7 with jasplakinolide. Data are representative of 4 independent experiments. Error bars in (B) and (C) represent standard errors.

During antigen recognition, TCR-pMHC complexes undergo a series of spatial translocations including: local clustering and long range transport to the center of the IS [6]–[10]. To explore the role of myosin IIA in these steps, we imaged fluorescently labeled TCRs at the cell-bilayer interface and tracked their movements with a custom tracking algorithm that implements an intensity gradient method to find centers of non-spherical fluorescent objects. Essentially, the entire ensemble of TCR microclusters within each individual cell (∼100 microclusters) was imaged and tracked with ∼50 nm spatial resolution and ∼50 ms temporal resolution over the course of IS formation. In control cells, TCR trajectories reveal coordinated centripetal movement in pSMAC region and the cell periphery following the initial cell-bilayer contact, but more confined motion at the center (cSMAC) (**Figure 1A**). Pharmacological inhibition of myosin IIA by blebbistatin (100 µM) and ML-7 (20 µM) does not alter the clustering of TCRs, but leads to much less directed motion of the microclusters (**Figure S1**). For a more quantitative measure, we analyzed the time-dependence of TCR translocation during IS formation. Averaged radial velocities, <V(*t*)>, are plotted against time, *t*, on a single cell basis. <V(*t*)> is defined negative for centripetal movements and positive for movements toward the cell periphery. In control cells, translocation of TCR microclusters varies significantly as IS formation proceeds (**Figure 1B**). After the initial cell-bilayer contact (*t* = 0 sec) microclusters undergo very rapid centripetal movement (<V(*t*)>_max_≈−70 nm/sec) for approximately 2 min and then maintain a reduced yet constant speed (<V(*t*)>≈−15 nm/sec) for an additional 3–5 min until the central accumulation of TCRs stabilizes. By contrast, TCR microclusters in cells pretreated with blebbistatin or ML-7 do not exhibit the rapid initial centripetal movement, but move at an almost constant velocity (<V(*t*)>≈−10 to −15 nm/sec) throughout the entire time course of IS formation (**Figure 1B**). The loss of the initial rapid component of centripetal movement indicates that myosin IIA is transiently involved in TCR transport and the slower movement in the presence of myosin inhibitors suggests a secondary driving force, presumably actin polymerization. In control experiments in which we simultaneously imaged TCR translocation and cell edge movement, we confirm that TCR microclusters, which move almost one order of magnitude faster than the cell membrane contraction (−5 nm/sec), are actively driven by forces from myosin IIA instead of the global cell movement (**Figure S2**).

Next, we quantified the effect of myosin IIA on actin retrograde flow during IS formation by imaging and tracking actin labeled with the calponin homology domain of utrophin fused to EGFP (EGFP-UtrCH) [20], [26]. Myosin IIA is known to exert contractile forces on the actin cytoskeleton for various cellular functions [27]–[30]. In agreement with previous reports [16], the flow of actin in control cells shows the same time-dependence as that of TCR microclusters: a rapid centripetal flow followed by a persistent and slower flow (**Figure 1C** and **Figure S3**). Similar to the effect of myosin inhibition on TCR translocation, actin flow in cells pretreated with ML-7 exhibits only a constant velocity of ≈−10 nm/sec. Blebbistatin was not used here due to its photoinactivation by short wavelength light [31]. Results from both TCR movement and actin flow indicate that myosin IIA transiently contributes to actin retrograde flow and, correspondingly, TCR transport during early times of IS formation. In T cells treated with both ML-7 (20 µM) and jasplakinolide (1 µM), a pharmacological agent to prevent actin depolymerization, actin retrograde flow is completely abrogated (**Figure 1C**). Therefore, actin polymerization provides a long-lasting driving force that operates in superposition to the more transient contribution from myosin.

**Figure 2:**
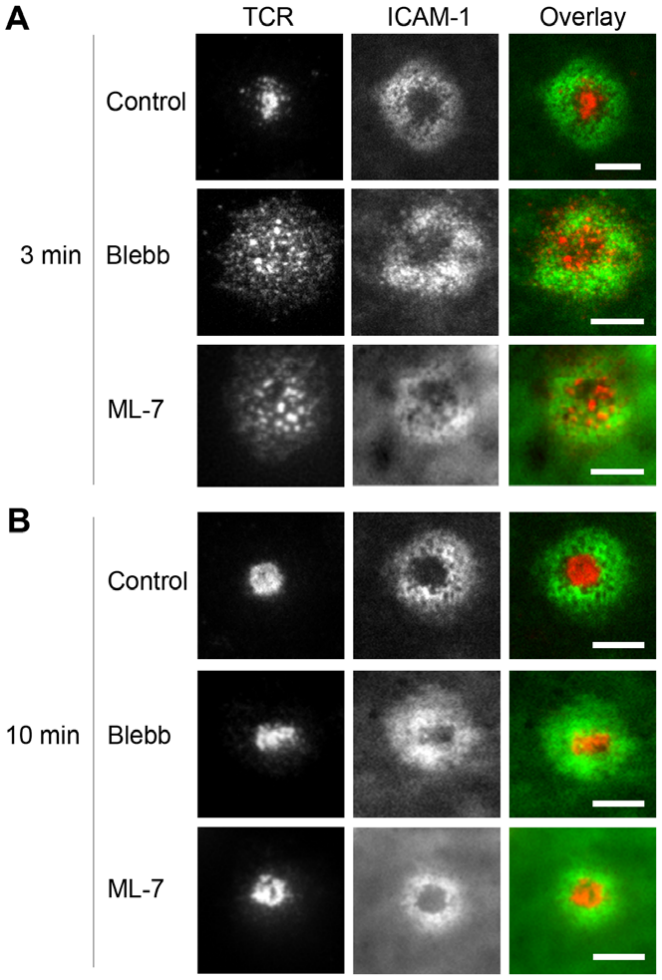
Inhibition of myosin only affects morphology of the early immunological synapse. Total internal reflection fluorescence (TIRF) images of TCRs labeled with H57 αTCR F_ab_ (Alexa Fluor 594) and ICAM-1 (Alexa Fluor 488) are shown. Cells were pretreated with DMSO, blebbistatin, or ML-7, and fixed at (A) 3 min and (B) 10 min after interacting with bilayers. Data are representative of 3 independent experiments. Scale bars: 5 µm.

The role of myosin IIA in IS formation has been controversial in previous studies [15], [23], [24]. Since our fluorescence tracking data suggests that TCR microclusters are able to translocate in the absence of myosin IIA, we studied whether myosin is required for the spatial organization at the IS. TCR and ICAM-1 in control cells exhibit the characteristic “bull's eye” pattern, where the TCR microclusters accumulate at the center while LFA-1 bound to ICAM-1 localizes at the periphery (**Figure 2**). Inhibition of myosin leads to a more dispersed distribution of TCR microclusters in cells fixed at 3 min after stimulation, but the difference becomes negligible when cells are fixed at 10 min (**Figure 2** and **Figure S4**). The overall ring-like distribution of ICAM-1 in the pSMAC is not affected by myosin inhibition. This demonstrates that myosin IIA influences the time frame of IS formation, but is not required for the superficial appearance of the final pattern. It is important to recognize that these observations do not suggest that the cells arrive at the same final state with and without myosin, as detailed below. Rather, it reveals that the large scale spatial organization of the IS probably offers insufficient information from which to judge the more subtle internal state of the cell.

### Forces applied to TCR clusters are translated to myosin IIA

**Figure 3:**
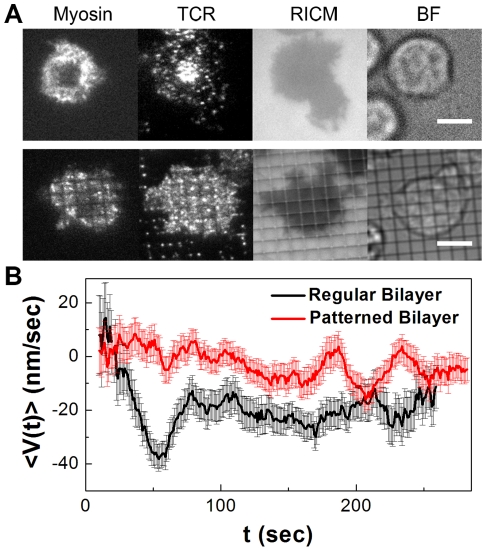
Physical constraints on TCR microcluster translocation impede myosin IIA movements. (A) TIRF, reflection interference contrast microscopy (RICM), and bright field (BF) images of T cells expressing EGFP-myosin on unpatterned or patterned bilayers. Scale bars: 5 µm. (B) Time-averaged radial velocities (<V(*t*)>) of EGFP-myosin in individual cells are plotted against the elapsed time (*t*) after the initial cell-bilayer contact (*t* = 0). Data are representative of 2 independent experiments.

Our results above have shown that myosin IIA transports TCR microclusters by driving actin retrograde flow. To further explore the mechanical link between myosin and TCR, we quantified if physical forces on TCR clusters can be transmitted to influence myosin. We formed lipid bilayers on substrates patterned with metal grids, which create barriers to lateral mobility of membrane-tethered pMHC and ICAM-1. Because patterned bilayers retain their fluidity [12], [17], [18], [20], [32], TCRs engaged with the constrained pMHC are trapped by the metal grids and experience passive opposing forces from the barriers that prevent their centripetal movement. As shown in **Figure 3A**, the adhesion of T cells and local clustering of TCRs remain unchanged on the patterned lipid bilayers, but TCR centripetal translocation is blocked by the metal barriers. The question, then, is how this physical trapping of TCR clusters affects myosin, which is itself not directly influenced by the substrate-imposed patterns. By plotting the radial velocity (<V(*t*)>) against time (*t*), we observed that myosin exhibits similar time-dependent motion as that of TCR and actin on non-patterned lipid bilayers. However, on a patterned bilayer where TCR microclusters are hindered from moving past the metal line grids, the centripetal movement of myosin IIA at earlier times is significantly reduced (**Figure 3B**). By performing the same experiments on actin, we also observed that actin retrograde flow decreases on the patterned lipid bilayers consistently with myosin (**Figure S5**). The slow-down of myosin in response to physically constrained TCRs confirms the existence of a mechanical coupling between TCR and myosin. Moreover, because contractile forces in actin are generated by myosin and studies have shown that resistive load on non-muscle myosin IIA leads to its slower power stokes on actin [33], the reduced actin retrograde flow on pattern lipid bilayers points to actin cytoskeleton as responsible for transmitting the resisting force from TCR microclusters to myosin.

### Myosin IIA is required for Ca^2+^ influx

**Figure 4:**
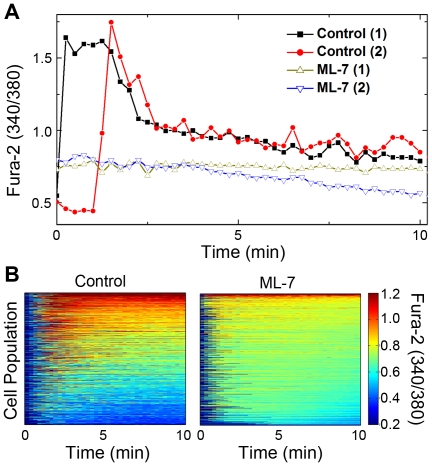
Inhibition of myosin IIA abolishes intracellular Ca^2+^ influx. The ratio of Fura-2 fluorescence emission intensity in response to 340 nm and 380 nm excitation (340/380) is proportional to intracellular [Ca^2+^]. (A) Fura-2 340/380 emission ratios are plotted against the cell stimulation time for four representative cells pretreated with either DMSO or ML-7. (B) Fura-2 340/380 emission ratios of control cells (n = 1602) and cells pretreated with ML-7 (n = 2187) are plotted against time on a color scale and organized along the y-axis according to the summed calcium influx.

A hallmark of T cell activation downstream of TCR signaling is the elevation of intracellular calcium, which in turn activates a number of calcium-dependent pathways [34]. We used Fura-2 as the indicator of intracellular calcium concentration; the ratio of its emission intensities at 340 nm excitation versus 380 nm is proportional to Ca^2+^ concentration. In control cells, intracellular Ca^2+^ concentration rapidly increases within 1 min after the initial cell-bilayer contact. By contrast, cells pretreated with ML-7 do not show any significant Ca^2+^ elevation, but maintain a low Ca^2+^ level similar to baseline (**Figure 4A**). The Ca^2+^ concentration traces of a large population of both control cells and cells pretreated with ML-7 show a dramatic reduction in the calcium influx in response to myosin IIA inhibition (**Figure 4B**). In agreement with previous work on Jurkat cells [24], the results show that myosin IIA is important for calcium signaling in primary T cells.

### Inhibition of myosin IIA reduces association of active ZAP-70 with TCR

**Figure 5:**
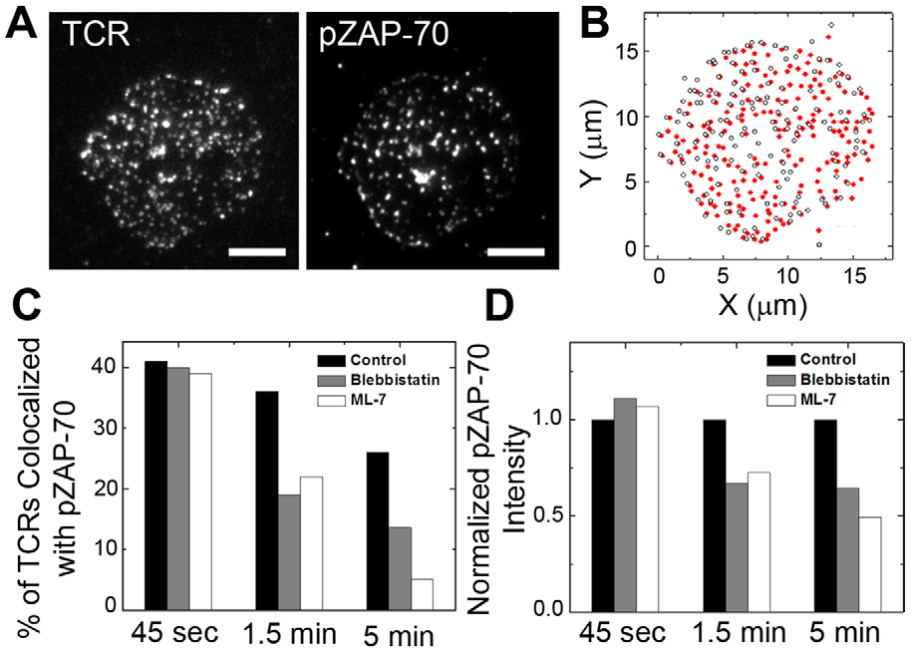
Inhibition of myosin IIA reduces phosphorylation of ZAP-70 and colocalization of pZAP-70 with TCR microclusters. (A) TIRF images of TCR and pZAP-70 (pY319) from a T cell fixed at 1.5 min. (B) Object-based colocalization analysis identifies TCRs and pZAP-70 in the T cell in panel (A). (C) The percentages of TCR microclusters colocalized with pZAP-70 are shown for the indicated stimulation times prior to fixation. (D) Fluorescence intensities of pZAP-70 in IS are shown normalized to those in control cells. Each column in panel (C) and (D) is an averaged value from approximately 200–300 cells. Data were reproduced in 2 independent experiments. Scale bars: 5 µm.

T cell signaling is initiated in discrete TCR microclusters, and association of kinases and adaptor proteins with the microclusters is a key step of sustaining the signaling reaction [9], [10], [34]–[36]. Upon TCR engagement to pMHC, ZAP-70 is recruited to the CD3 zeta chain and phosphorylated on tyrosine 319 (pZAP-70) for downstream signaling. To understand the role of myosin IIA in initiation of TCR signaling, we used ZAP-70 as a quantitative readout of TCR/CD3 signaling and quantified how myosin inhibition influences colocalization of pZAP-70 with TCR using an object-based colocalization algorithm. Unlike many intensity-based colocalization algorithms [37], this analysis avoids bias due to the variation in fluorescence intensities between TCR microclusters (**Figure 5B**). In control cells, pZAP-70 localizes mainly on the cell periphery and its colocalization with TCR decreases with stimulation time (**Figure 5A and C** **, Figure S6**). Inhibition of myosin IIA results in less colocalization of TCR microclusters with pZAP-70 in T cells fixed at both 1.5 min and 5 min after stimulation, but not at 45 sec (**Figure 5C**). The phosphorylation level of ZAP-70 at the IS also decreases in the absence of myosin function (**Figure 5D**). Myosin IIA therefore contributes to the stable association of active ZAP-70 with TCR, but it is not necessary for the initial recruitment.

### Inhibition of myosin IIA reduces CasL phosphorylation

To our knowledge, there have been no previous studies directly quantifying the cytoskeletal strain in T cells and correlating this to TCR signaling. The data we report above and other published results clearly indicate at least an indirect influence of myosin IIA on TCR signaling, but the role of its mechanical forces in the process still remains unclear. To explore that question we studied the phosphorylation of CasL. It is a member of the mechanosensing Cas protein family and is predominately expressed in T lymphocytes [38]–[41]. All Cas family proteins contain a highly conserved Src kinase substrate domain, which is consisted of multiple Tyr-x-x-Pro (YxxP) motifs. Studies on p130Cas, one of the Cas proteins, have shown that mechanical stretching changes conformation of the motifs and leads to enhancement of tyrosine phosphorylation and possibly downstream signaling [42]. Whether or not CasL is involved in molecular force transduction is less clear, but its phosphorylation level strongly depends on actin integrity in several cell types [43]–[45], suggesting that CasL might also function as a mechanosensor. Therefore, we investigated if CasL plays a possible role to transduce myosin contractile forces to modulate TCR signaling.

**Figure 6:**
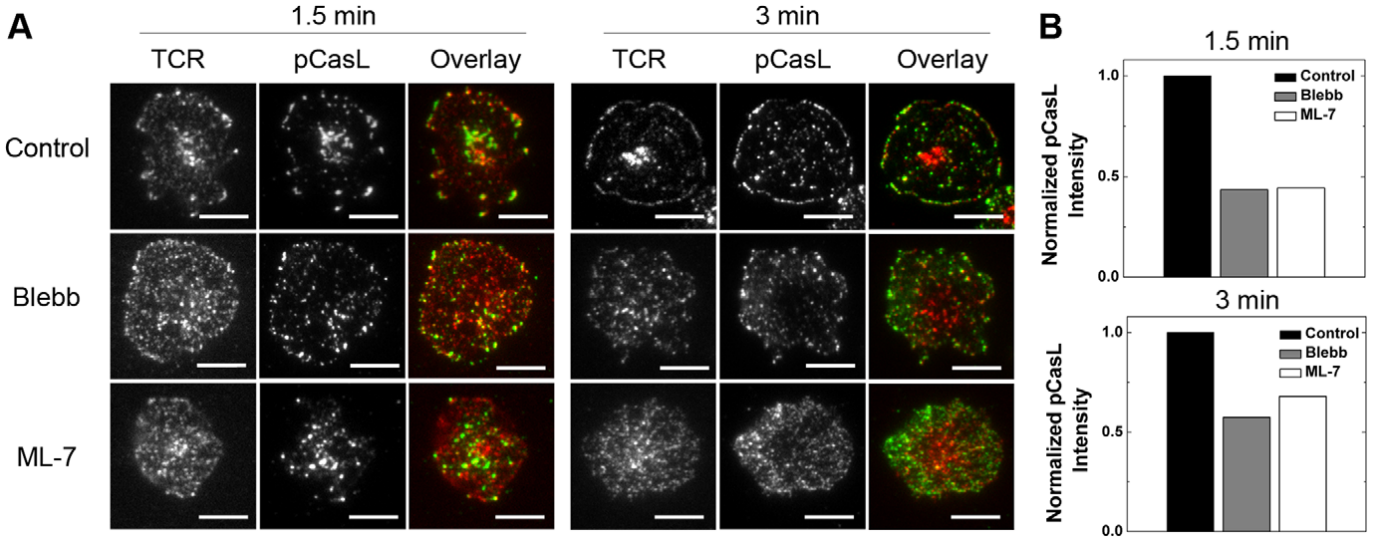
Inhibition of myosin IIA reduces phosphorylation of CasL. (A) TIRF images of TCR and pCasL from T cells fixed at 1.5 min and 3 min and pretreated with DMSO (control), blebbistatin or ML-7. (B) Fluorescence intensities of pCasL in IS are shown normalized to those in control cells. Each column in Panel (A) and (B) is an averaged value from approximately 200 cells. Data were reproduced in 2 independent experiments. Scale bars: 5 µm.

We quantified the phosphorylation of CasL by using a phosphorylation-specific antibody against its YxxP motifs and measuring the fluorescence intensity of secondary antibodies at the IS. It has been previously reported that either TCR or integrin crosslinking leads to CasL phosphorylation through possibly independent signaling pathways, and that the phosphorylation level upon TCR ligation peaks transiently within the first 5 min after stimulation while integrin crosslinking results in a later but more long-lasting phosphorylation [38]. Because our entire study here focuses on the early events of T cell signaling as well as the relevance between TCR and myosin, only pMHC was present in the supported lipid bilayer to exclude the potential influence of integrin signaling pathways on CasL function. While the nature of the IS is somewhat different without ICAM-LFA interactions, T cells can adhere and be normally activated by bilayer-tethered pMHC alone [46]. As shown in **Figure 6A**, phosphorylated CasL (pCasL) in control cells colocalizes with TCR microclusters across the entire IS at 1.5 min after stimulation. However, at a later time point (t = 3 min), pCasL is absent from the cSMAC region but still colocalizes with the discrete TCR microclusters on the cell periphery. Inhibition of myosin IIA has negligible effects on colocalization of TCR microclusters with pCasL, quantified by using the object-based colocalization analysis (**Figure S7**). However, immunofluorescence quantification of pCasL at the IS shows significantly reduced phosphorylation of CasL by myosin inhibition at both 1.5 min and 3 min after stimulation (**Figure 6B**).

## Discussion

It is increasingly clear that the ability of cells to sense, interpret, and respond to mechanical signals plays a critical role in modulating diverse cellular functions, such as proliferation, migration, differentiation and homeostasis [47]–[50]. While integrins are the well-known force transducers in cells, recent data suggest that membrane receptors that are not directly associated with focal adhesions may also couple into force sensing roles, at least indirectly [2]. In T cells, the concept of force sensing is not well established although a number of recent studies have suggested the idea of mechanosensing in T cell activation [51]–[53]. We suggest that an indirect role for force in TCR signaling is all but guaranteed by the known significance of spatial organization in this system [12]–[14], [17]–[20]. Any applied force that changes protein spatial organization in a manner to impact signaling reactions affords an indirect force response to the system. The resulted signaling, however, may be either reduced or enhanced depending on the exact mechanism. Previous studies have reported that impeded translocation of TCR microclusters leads to enhanced signaling, likely due to attenuated signal degradation at cSMAC [12], [22], [35]. By contrast, our results, in agreement with a previous study [24], suggest that inhibition of myosin leads to slower TCR transport and diminishes signaling. The bigger question is whether force from myosin plays a direct role in the modulation of TCR signaling. Although actin, microtubule, and some molecular motors have all been shown to play important mechanical roles in T cell signaling [10], [15], [16], [22], [24], they are unlikely to directly transduce mechanical forces into biochemical signaling cascades. Our observation of a decrease in CasL phosphorylation in response to myosin inhibition suggests that CasL may be involved in a mechanical signal transduction process in T cells. While working out details of the possible regulatory pathways is well beyond the scope of this paper, CasL is clearly a candidate for relating myosin to TCR signaling pathways. Studies have shown that CasL may be a substrate for Fyn and Lck, two key tyrosine kinases in initiating TCR activation [41]. Phosphorylated CasL can also bind to Src homology (SH) domains of signaling proteins, such as Crk, Cbl, and nucleotide exchange protein C3G, to regulate T cell signaling [54], [55]. We observed the phosphorylation of CasL upon TCR ligation and its association with discrete TCR microclusters at the IS. Our results of calcium influx, ZAP-70 phosphorylation, and TCR microcluster formation all suggest that myosin is more important for sustained signaling than initiation. CasL might be involved in a feedback loop between myosin and multiple signaling pathways. While much remains to be uncovered concerning the nature of mechanical influences on TCR activation, our observation of differential CasL phosphorylation with myosin inhibition clearly pinpoints a starting point to look into.

## Materials and Methods

### Ethics statement

All animal work was conducted with prior approval by Lawrence Berkeley National Laboratory Animal Welfare and Research Committee (AWRC) and performed under the approved protocol 17702.

### Animals

AND X B10.BR transgenic mice (Jackson Laboratory), of both genders and of age between 6–16 weeks, were used as CD4+ cell donors. Mice were housed in a facility certified by AWRC, under continuous veterinary animal care with adequate water, food and comfort. Only AWRC veterinary certified researchers, who have passed specific animal handling tests for the procedure, were allowed to handle the mice.

### CD4+ cell harvest

The procedure was performed in accordance with the American Veterinary Medical Association (AVMA) Guidelines on Euthanasia. Mice were first euthanized with carbon dioxide. Cervical dislocation was performed at least 5 minutes after euthanasia to minimize pain to the mice. Mice were sterilized with 70% ethanol prior to the harvest of lymph nodes and spleen. AND CD4+ T cells were expanded to T cell blasts after harvest and maintained as previously described [12], [56].

### DNA constructs

A plasmid containing enhanced green fluorescent protein fused to the calponin homology domain of utrophin (EGFP-UtrCH) was a gift of Dr. William Bement, University of Wisconsin, Madison, WI. The EGFP-UtrCH coding sequence was amplified using PCR and subcloned into a murine stem cell virus plasmid (pMSCV-Puro). A plasmid containing EGFP fused to the heavy chain of human non-muscle myosin IIA (EGFP-NMHCIIA) was provided by Dr. Robert Adelstein, National Institutes of Health, Bethesda, MD through Addgene.org (Addgene plasmid 11347) [57], and the EGFP-NMHCIIA coding sequence was subcloned into pMSCV-Puro plasmid.

### Reagents

Histidine-tagged ICAM-1 and MHC Class II I-E^K^ were expressed and purified as previously described [12], [58]. Briefly, secreted ICAM-1 with a decahistidine tag at its C terminus (a gift of Dr. Mark Davis, Stanford University) was expressed using the baculovirus expression system in High Five cells (Invitrogen) and purified using a Ni^2+^-NTA-agarose column (Qiagen). Secreted MHC with a hexahistidine tag at the C terminus of both α and β chains was similarly expressed and purified from S2 cells. Blebbistatin, ML-7 and jasplakinolide were purchased from EMD Chemicals. ZAP-70 (Tyr319) antibody and p130Cas (pY165) antibody were purchased from Cell Signaling.

### Retroviral transfection

T cells were retrovirally transduced using supernatants derived from cultures of Phoenix cells as previously described [59]. Briefly, Phoenix cells were transfected with pMSCV-Puro-EGFP-UtrCH or pMSCV-Puro-EGFP-NMHCIIA immediately prior to T cell harvest using the calcium phosphate method. The transfected Phoenix cells were cultured in T cell medium starting 24 hours after transfection and T cell harvest. Two days after transfection and T cell harvest, T cells were spun down, resuspended in supernatant collected from the Phoenix cell cultures, and spun at 2500 min^−1^ for 1 hr to encourage uptake of virus. T cells were selected in fresh medium containing 0.5 ug/ml puromycin 3 days after harvest and were allowed to recover from selection in fresh medium 5 days after harvest. They were used for imaging experiments starting from day 7 after cell culture.

### Bilayer assembly and cell imaging

Moth cytochrome *c* 88–103 peptide (MCC, ANERADLIAYLKQATK) (Biosynthesis and the Dana-Farber Core Facility) was loaded onto the I-E^K^ protein overnight. Glass-supported lipid bilayers comprised of 98 mol% 1,2-dioleoyl-sn-glycero-3-phosphocholine (DOPC) and 2 mol% 1,2-dioleoyl-sn-glycero-3-[(N-(5-amino-1-carboxypentyl) iminodiacetic acid) succinyl] (nickel salt) (Ni^2+^-NTA-DOGS) (Avanti Lipids) were prepared in flow chambers by standard methods [12], [14], [19]. Bilayers were loaded with ICAM-1 and pMHC by incubation at room temperature for 40 minutes. To label the T cell receptors, the non-blocking antibody H57 αTCR-F_ab_ (conjugated to Alexa Fluor 594 or Alexa Fluor 643 as indicated) was incubated with T cells at 4°C for 20 min. To inhibit myosin or actin depolymerization, cells were then incubated with 100 µM blebbistatin, 20 µM ML-7 or 1 µM jasplakinolide at 37°C for 15 min before imaging. Inhibitors used at the indicated concentrations have been shown to effectively inhibit functions of their target proteins [60]–[62]. For fixed cell experiments, cells were injected into a sample chamber preheated to 37°C and allowed to interact with the bilayer for the indicated time. Cells were then fixed with 2% paraformaldehyde, permeabilized with 0.05% Triton X, blocked with 5% casein and labeled with antibodies at room temperature or as otherwise indicated. To label pZAP-70, fixed cells were incubated with anti-pZAP-70 IgG against Tyr319 (Cell Signaling) at 4°C overnight and then with Alexa Fluor-488 conjugated goat anti-rabbit IgG (Invitrogen) at room temperature for 20 min. Because the YxxP motifs are highly conserved between CasL and p130Cas, the YxxP motifs of CasL were labeled with p130Cas (pY165) antibody (Cell Signaling). Total internal reflection fluorescence (TIRF) microscopy images were acquired on a Nikon Ti-E/B inverted microscope with a 100× 1.49 NA oil immersion TIRF objective and an Andor iXon EMCCD camera. Images of phosphorylated pZAP70 or pCasL of different samples were acquired under the exact same settings for lasers, camera, and microscope; the angle of the input laser was also kept consistent throughout imaging under the control of a motorized laser TIRF illumination unit (Nikon) and the focus on the glass substrate was maintained by a Perfect Focus System (Nikon).

### Calcium imaging

T cells were firstly incubated with 1 µM Fura-2-acetoxymethyl ester (Fura-2 AM) in serum-free cell media at room temperature for 15 minutes and then in Fura-2 free serum-rich media at 37°C for 20 minutes. After TCR labeling, cells were incubated with DMSO or 20 µM ML-7 at 37°C for 15 min before being injected into the imaging chamber. Images were acquired on a Nikon TE 2000 microscope with a 40× S Fluor objective (Nikon) and a Coolsnap K4 camera (Roper Scientific). Emission at 510 nm was captured by alternating the excitation wavelength between 340 and 380 nm. The ratiometric value of the Fura-2 AM dye, indicating relative intracellular calcium levels, was obtained by using the program Imaris (Bitplane) and a custom Matlab algorithm.

### Speckle tracking and image analysis

The custom speckle tracking algorithm as described previously [18], [20], was used to identify the locations of speckles based on the fluorescent intensity gradient within the images. Following identification, nearest neighbors of speckles in consecutive frames were linked to generate trajectories. The position- and time- averaged radial velocities of the speckles relative to the defined cell center were then analyzed. Time-averaged radial velocity <V(*t*)> was obtained by averaging the radial velocities of all microclusters located at the cell periphery during (*t*, *t*+Δ*t*), where *t* is the elapsed time after the initial cell-bilayer contact and Δ*t* = 10 sec. The object-based colocalization algorithm contains two major steps: cluster identification and pairwise cluster matching between two fluorescent channels. The fluorescent clusters were firstly identified by the speckle tracking algorithm. The microclusters in two fluorescent channels are considered as colocalized if their center distance is within the diffraction limit (200 nm). TCRs accumulated in the cSMAC area were excluded from the analysis for control cells at 5 min due to their distinct signaling properties and the absence of ZAP-70 in this region [9]. The cSMAC region was outlined based on the higher fluorescence intensity by applying an imaging threshold calculated from Otsu's algorithm [63]. For the direct comparison of the phosphorylation level of pZAP-70 or pCasL between different samples, analysis was done in multiple steps. (1) Cells were first selected manually and outlined in each image. (2) Fluorescence intensities of all pixels at each cell-bilayer contact area were summed and an averaged fluorescence intensity per cell (I_ave_) subtracted by background was obtained. (3) I_ave_ of each sample was then normalized to that of the control.

## Supporting Information

Figure S1 **Inhibition of myosin IIA affects centripetal transport of TCR microclusters.** (A) TCRs were labeled with H57 αTCR F_ab_ (Alexa Fluor 594) and imaged starting from the initial cell-bilayer contact (*t* = 0 sec). All trajectories of TCR microclusters in one representative cell pretreated with ML-7 are shown. Color bar corresponds to the elapsed time after the initial cell-bilayer contact. Scale bar: 5 µm (B) Magnified images of trajectories located at the central and peripheral areas of the immunological synapse.
(TIF)

Figure S2 **Simultaneous tracking of TCR microclusters and cell edge movement.** (A) Time-averaged radial velocities, <V(*t*)>, of all TCR microclusters in a control cell are plotted against the elapsed time after the initial cell-bilayer contact. (B) Cell radii obtained from RICM images are plotted against time. The average spreading and contraction velocities, 95 nm/sec and −5 nm/sec, respectively, were calculated by linear fitting. (C) The relative velocities of TCR microclusters, calculated by subtraction of cell edge movement from TCR radial velocities at each time point, are plotted against time.
(TIF)

Figure S3 **TCR translocation is coupled to actin retrograde flow.** (A) Simultaneous total internal reflection fluorescence (TIRF) images of TCR labeled with αTCR F_ab_ (Alexa Fluor 594) and EGFP-UtrCH during the formation of an immunological synapse in a control cell. (B) Time-averaged radial velocities, <V(*t*)>, of all TCR microclusters and EGFP-UtrCH in control cells and cells pretreated with ML-7 are plotted against the elapsed time (*t*) after the initial cell-bilayer contact. Scale bars: 5 µm.
(TIF)

Figure S4 **Morphological quantification of the immunological synapse.** TIRF images of TCRs labeled with H57 αTCR Fab (Alexa Fluor 594) and ICAM-1 (Alexa Fluor 488) are shown. Cells were pretreated with DMSO, blebbistatin, or ML-7, and fixed at (A) 3 min and (B) 10 min after interacting with bilayers. Normalized intensities of TCRs and ICAM-1 are plotted versus the radial distance from the center of the immunological synapse. Data are representative of 3 independent experiments. Scale bars: 5 µm.
(TIF)

Figure S5 **Physical constraints on TCR microcluster translocation impede actin retrograde flow.** (A) TIRF, reflection interference contrast microscopy (RICM), and bright field (BF) images of T cells expressing EGFP-UtrCH on unpatterned or patterned bilayers. Scale bars: 5 µm. (B) Time-averaged radial velocities (<V(*t*)>) of EGFP- UtrCH in individual cells are plotted against the elapsed time (*t*) after the initial cell-bilayer contact. Data are representative of 2 independent experiments.
(TIF)

Figure S6 **TIRF images of TCR and pZAP-70 (pY 319) in T cells pretreated with DMSO, blebbistatin, or ML-7. T cells were fixed at the time indicated after the initial cell-bilayer contact.**
(TIF)

Figure S7 **Colocalization of TCR and pCasL in T cells pretreated with DMSO, blebbistatin, or ML-7.** The percentages of TCR microclusters colocalized with pCasL are shown for the indicated stimulation times prior to fixation. Each column is an averaged value from approximately 200 cells. Data were reproduced in 2 independent experiments.
(TIF)
