# Supplementary figures and images for: Myosin IIA Modulates T Cell Receptor Transport and CasL Phosphorylation during Early Immunological Synapse Formation
Source: PLoS One. 2012 Feb 8;7(2):e30704. doi: 10.1371/journal.pone.0030704 (PMC3275606; doi:10.1371/journal.pone.0030704)

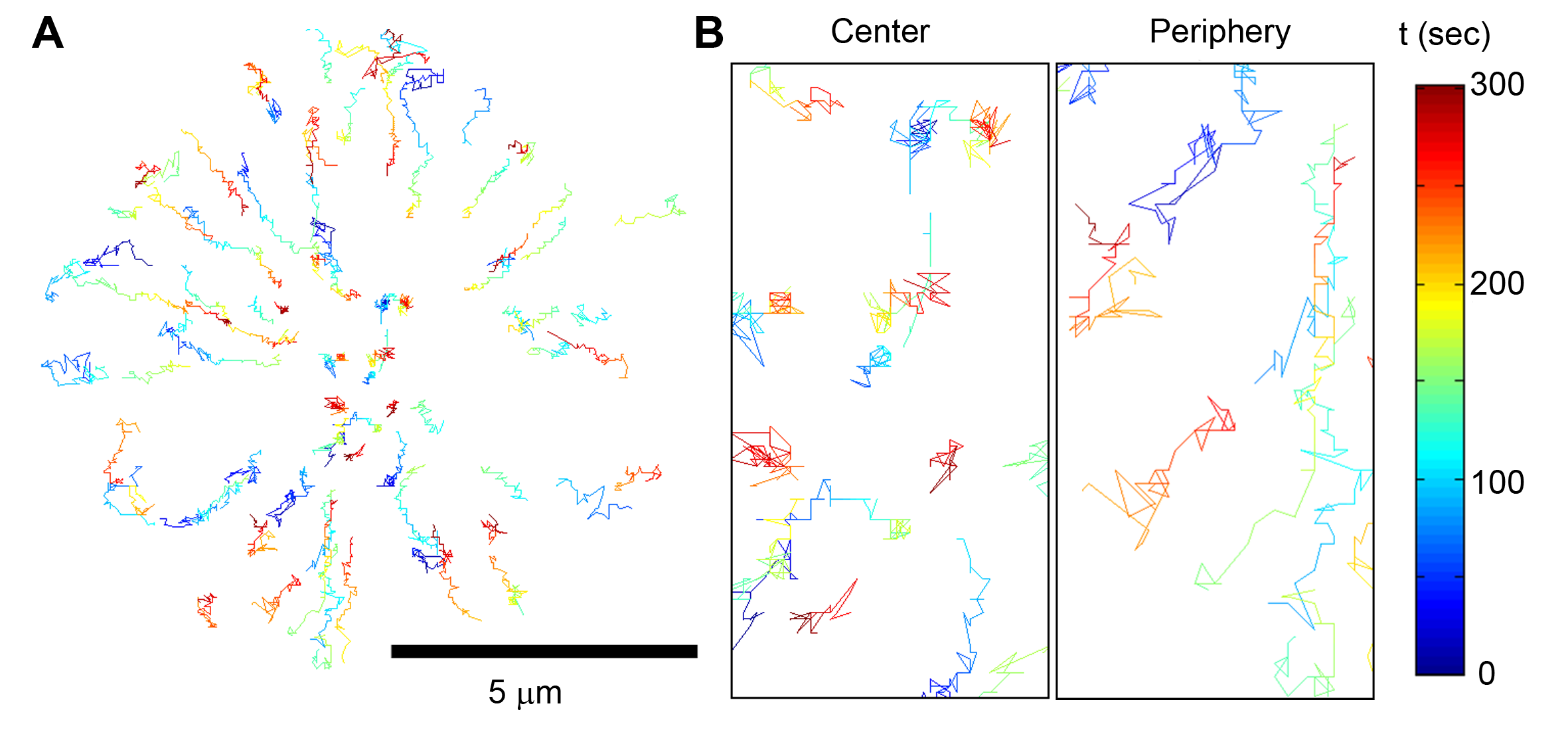

Supplement: Figure S1 — Inhibition of myosin IIA affects centripetal transport of TCR microclusters. (A) TCRs were labeled with H57 αTCR Fab (Alexa Fluor 594) and imaged starting from the initial cell-bilayer contact (t = 0 sec). All trajectories of TCR microclusters in one representative cell pretreated with ML-7 are shown. Color bar corresponds to the elapsed time after the initial cell-bilayer contact. Scale bar: 5 µm (B) Magnified images of trajectories located at the central and peripheral areas of the immunological synapse. (TIF) [file pone.0030704.s001.tif]

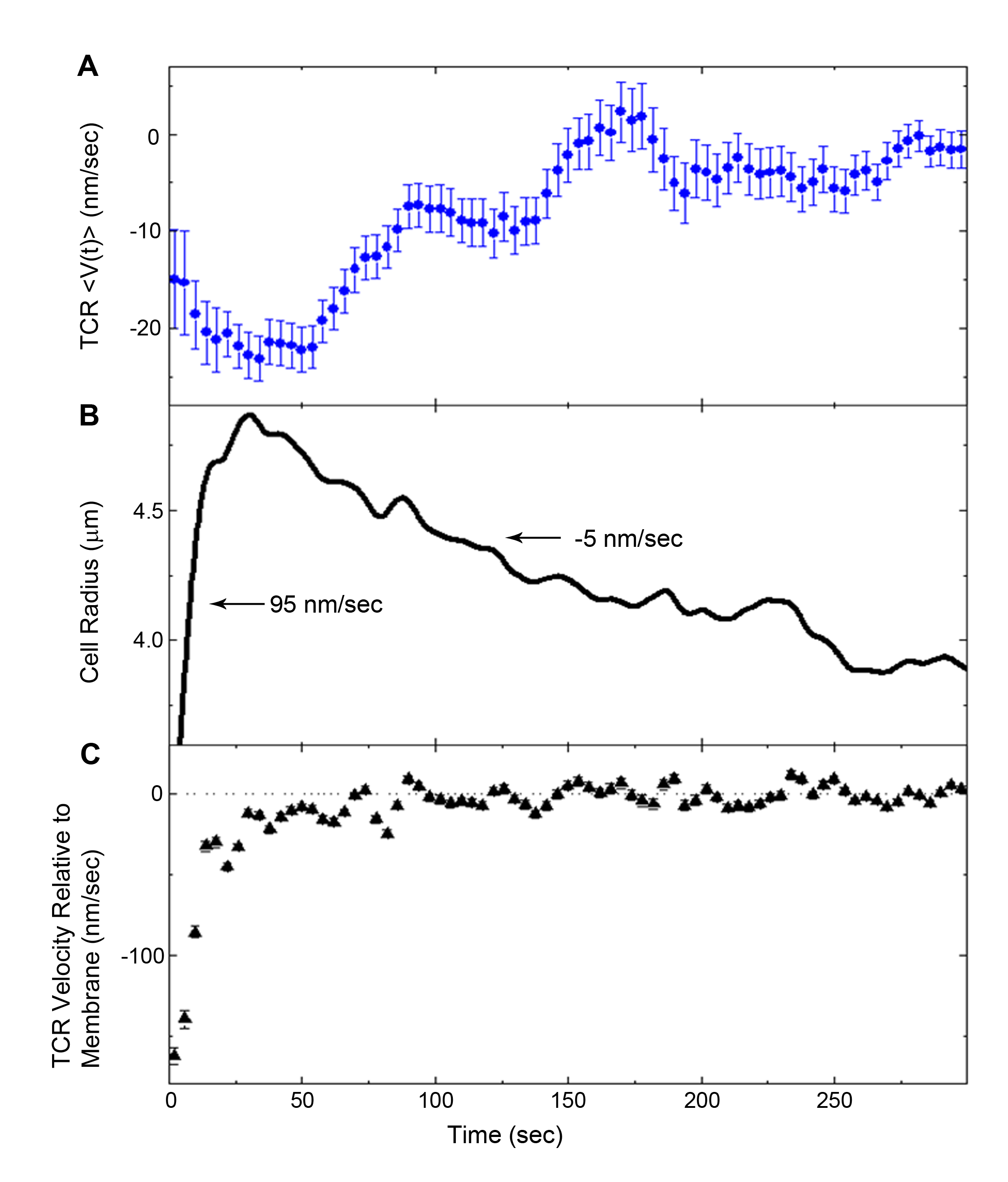

Supplement: Figure S2 — Simultaneous tracking of TCR microclusters and cell edge movement. (A) Time-averaged radial velocities, <V(t)>, of all TCR microclusters in a control cell are plotted against the elapsed time after the initial cell-bilayer contact. (B) Cell radii obtained from RICM images are plotted against time. The average spreading and contraction velocities, 95 nm/sec and −5 nm/sec, respectively, were calculated by linear fitting. (C) The relative velocities of TCR microclusters, calculated by subtraction of cell edge movement from TCR radial velocities at each time point, are plotted against time. (TIF) [file pone.0030704.s002.tif]

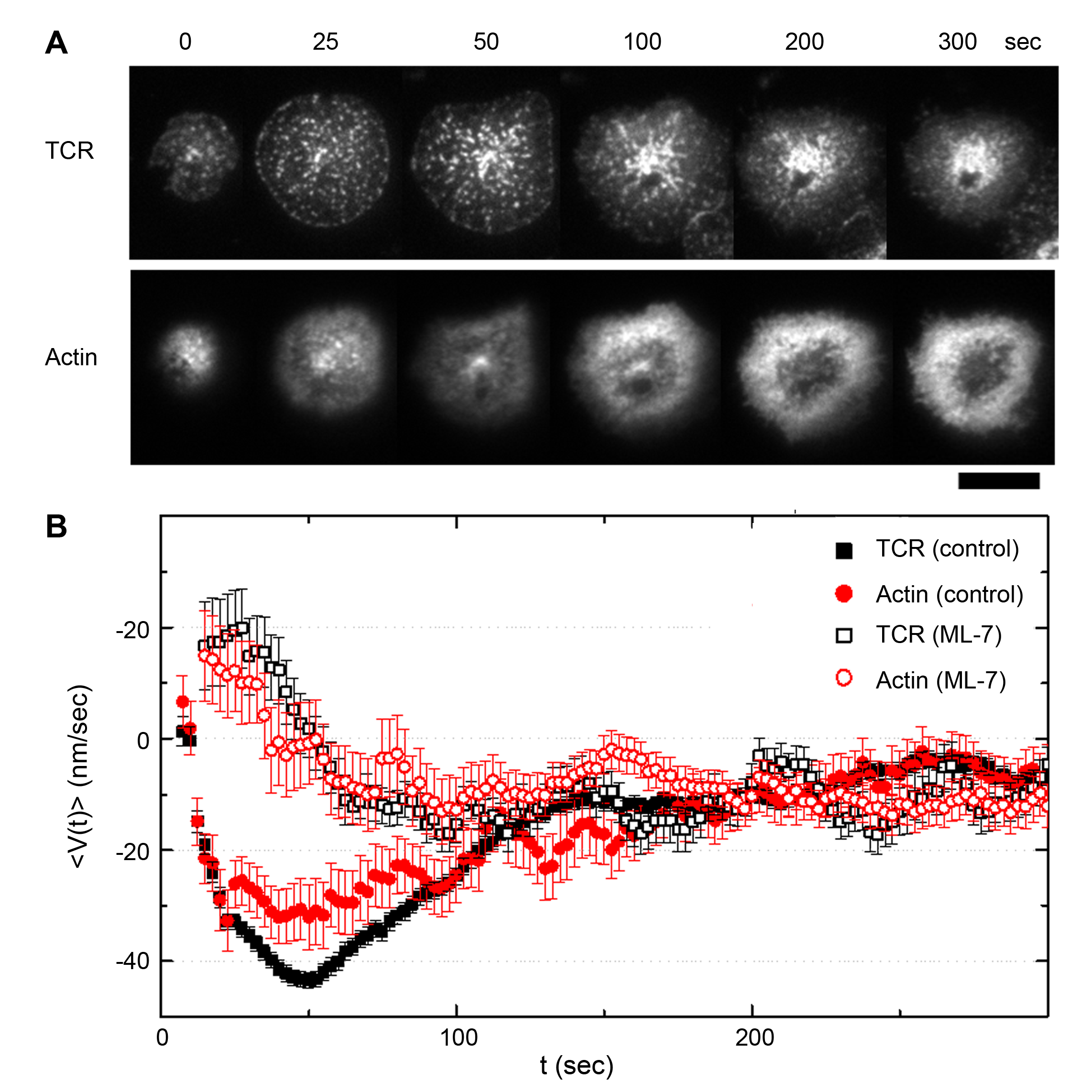

Supplement: Figure S3 — TCR translocation is coupled to actin retrograde flow. (A) Simultaneous total internal reflection fluorescence (TIRF) images of TCR labeled with αTCR Fab (Alexa Fluor 594) and EGFP-UtrCH during the formation of an immunological synapse in a control cell. (B) Time-averaged radial velocities, <V(t)>, of all TCR microclusters and EGFP-UtrCH in control cells and cells pretreated with ML-7 are plotted against the elapsed time (t) after the initial cell-bilayer contact. Scale bars: 5 µm. (TIF) [file pone.0030704.s003.tif]

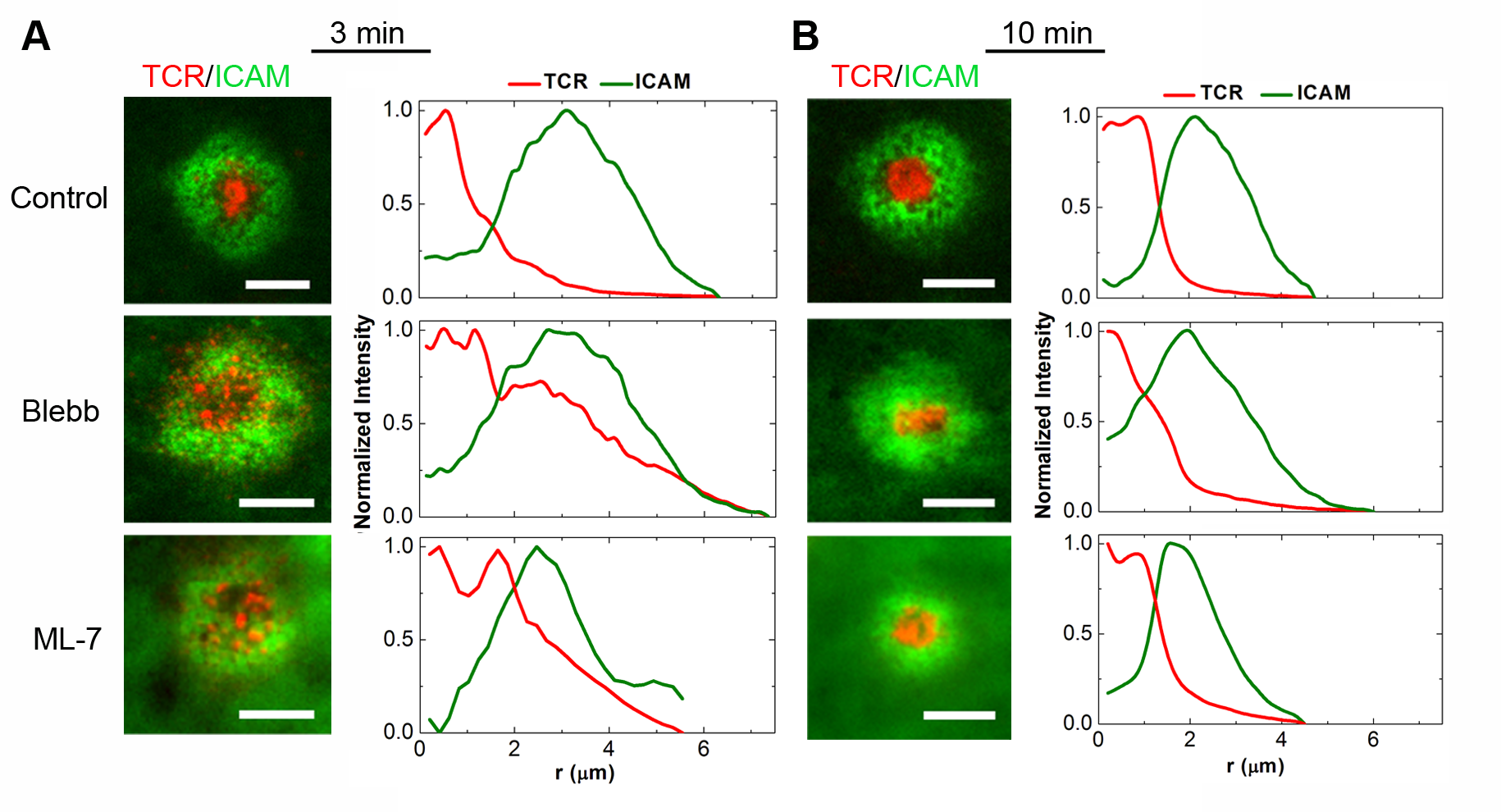

Supplement: Figure S4 — Morphological quantification of the immunological synapse. TIRF images of TCRs labeled with H57 αTCR Fab (Alexa Fluor 594) and ICAM-1 (Alexa Fluor 488) are shown. Cells were pretreated with DMSO, blebbistatin, or ML-7, and fixed at (A) 3 min and (B) 10 min after interacting with bilayers. Normalized intensities of TCRs and ICAM-1 are plotted versus the radial distance from the center of the immunological synapse. Data are representative of 3 independent experiments. Scale bars: 5 µm. (TIF) [file pone.0030704.s004.tif]

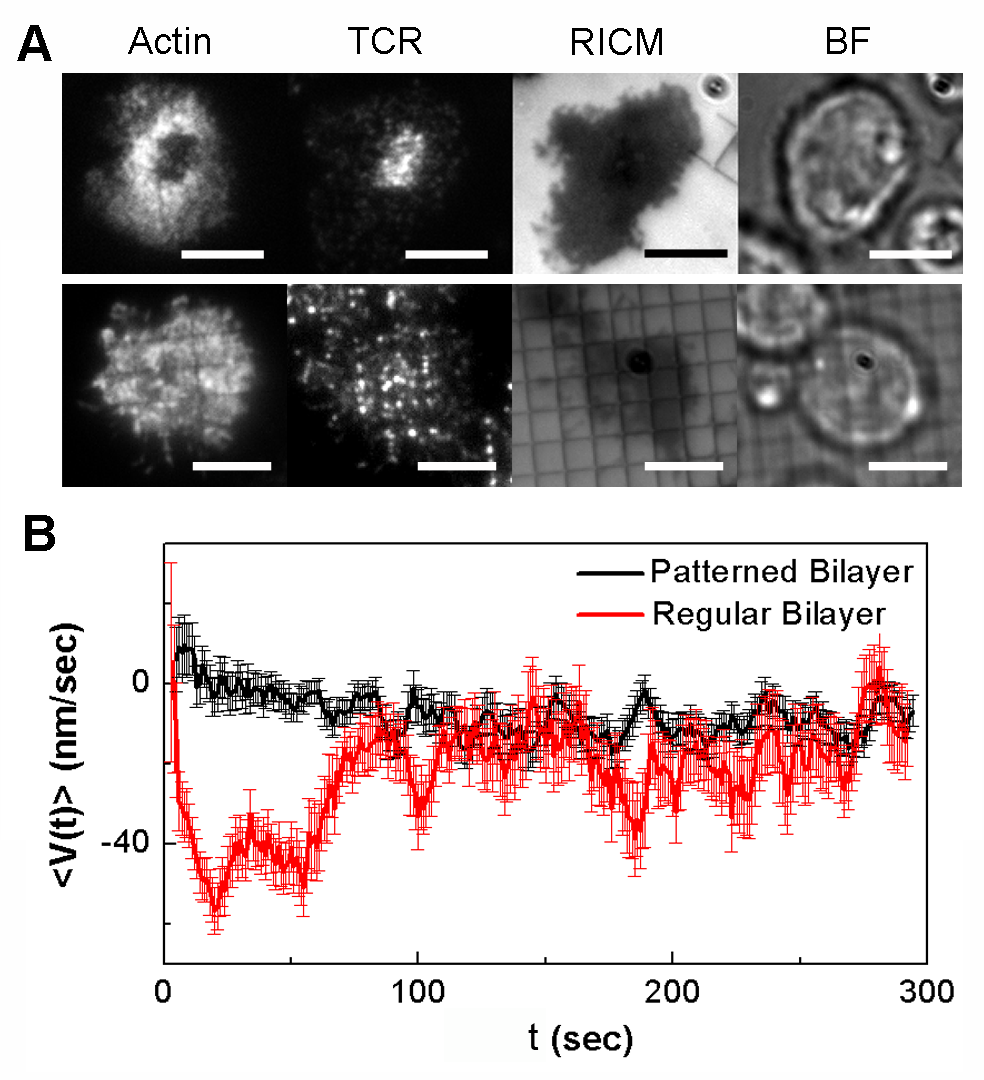

Supplement: Figure S5 — Physical constraints on TCR microcluster translocation impede actin retrograde flow. (A) TIRF, reflection interference contrast microscopy (RICM), and bright field (BF) images of T cells expressing EGFP-UtrCH on unpatterned or patterned bilayers. Scale bars: 5 µm. (B) Time-averaged radial velocities (<V(t)>) of EGFP- UtrCH in individual cells are plotted against the elapsed time (t) after the initial cell-bilayer contact. Data are representative of 2 independent experiments. (TIF) [file pone.0030704.s005.tif]

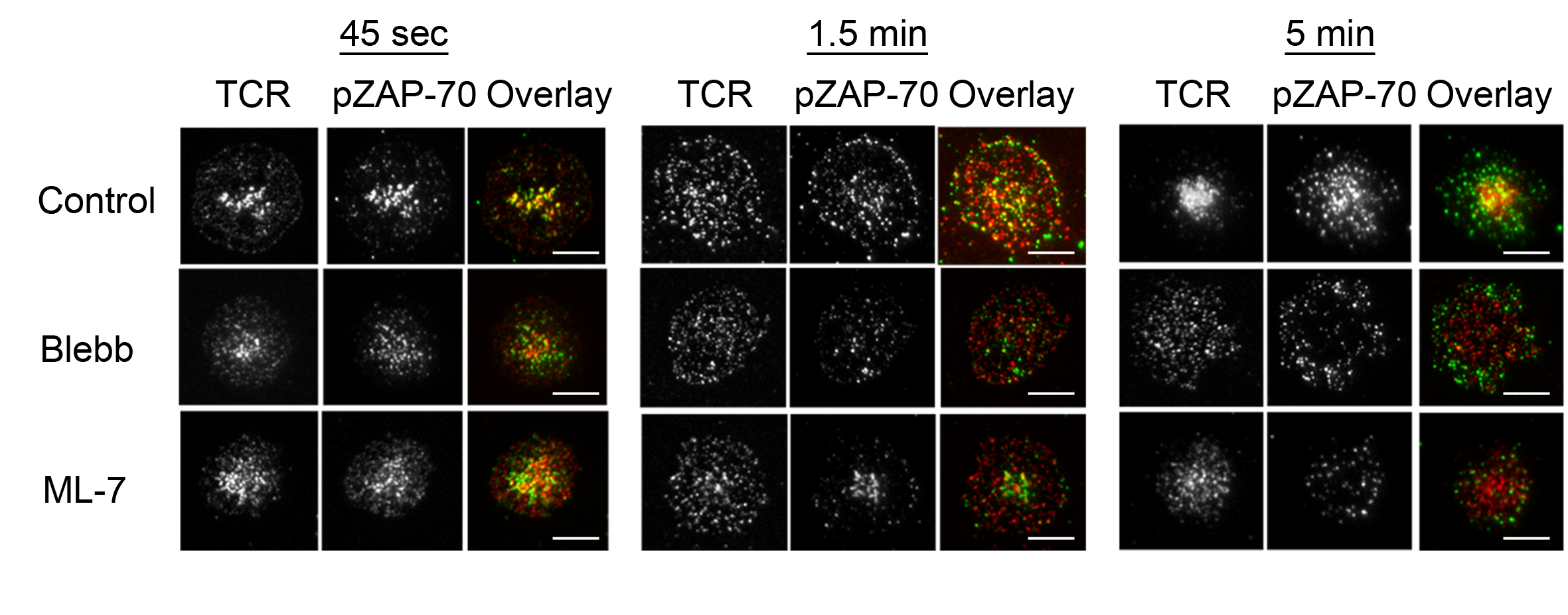

Supplement: Figure S6 — TIRF images of TCR and pZAP-70 (pY 319) in T cells pretreated with DMSO, blebbistatin, or ML-7. T cells were fixed at the time indicated after the initial cell-bilayer contact. (TIF) [file pone.0030704.s006.tif]

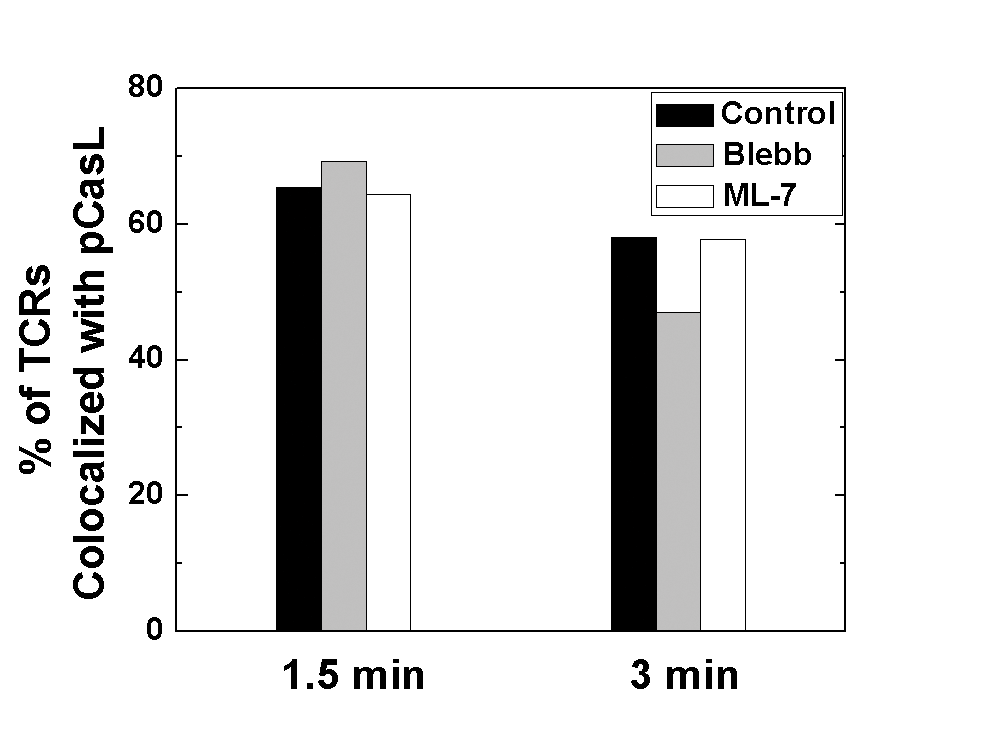

Supplement: Figure S7 — Colocalization of TCR and pCasL in T cells pretreated with DMSO, blebbistatin, or ML-7. The percentages of TCR microclusters colocalized with pCasL are shown for the indicated stimulation times prior to fixation. Each column is an averaged value from approximately 200 cells. Data were reproduced in 2 independent experiments. (TIF) [file pone.0030704.s007.tif]
